# Subjective wet perception assessment of fabrics with different drying time

**DOI:** 10.1098/rsos.180798

**Published:** 2018-08-15

**Authors:** Kam-Hong Chau, Ka-Po Maggie Tang, Chi-Wai Kan

**Affiliations:** Institute of Textiles and Clothing, Hong Kong Polytechnic University, Hung Hom, Hong Kong

**Keywords:** wet perception, drying, evaporation, fabric, subjective assessment, comfort

## Abstract

Wet perception involves a complex neurobiological mechanism and it is a crucial factor affecting the wear comfort in daily life. A subjective wet perception assessment was conducted against wetted fabrics. The assessment method was set to demonstrate the sensation felt by the wearer in recovery period after light activities, and assumes that there is no further sweat secretion. Twenty participants participated in the assessment. Participants were presented with fabrics dried with different duration for simulating garments dry during recovery period. A new fabric driver was built to simulate body movements during wear. The driver drove specimens and reference fabrics on participants' forearms. The two-arm configuration of the fabric driver helps to enhance the reliability of assessment results. The participants were asked to give wetness rating on each sample in ratio scale. We conclude that log_10_ of subjective wetness rating has linear relationship with drying time of fabric (DToF) and amount of water in fabric. A novel wetness factor (WF) is developed to quantify the effects of wet perception and exposure time induced by a drying fabric. WF is the area under curve of wetness rating against DToF. A smaller WF indicates that a user suffers less from wet sensation.

## 1. Introduction

Human skin has no hygroreceptor [1–3], but wet perception can be evoked by thermoreceptors and mechanoreceptors [4–8]. In general, cool stimuli could trigger wet perception, and they give stronger wet perception than warm stimuli [4,7,8]. If cool stimulus is absent, mechanical signal may contribute more to the wet perception [9]. However, the mechanism of wet perception by the receptors is complicated and not clear until now [9–11]. On the other hand, drying of fabric refers to evaporation of water from wetted fabric, and the fabric is commonly wetted by sweating. For example, a shirt is wetted by sweat when a wearer walks under sunshine or in hot weather. The wearer may not have time to change clothes before going out for a meeting or dining out. In such cases, the wearer may experience uncomfortable post-exercise chill sensation [12–15]. The shirt gives a wet sensation to the wearer for an extended period of time before it turns dry. In addition to wet perception, water at skin–fabric interface will increase friction or adhesion at the interface [16–19]. When there is prolonged contact between skin and wetted fabric, skin injuries can be induced [20,21].

In addition to the time dependency of wet perception, the amount of water carried in fabric has correlation with wet perception. Raccuglia *et al*. [22,23] have found that wetness perception is related to fabric saturation values (amount of moisture per unit volume of fabric). Another study [18] has indicated that under dynamic skin–fabric contact, surface roughness of fabric contributes to reduce wetness sensation. Both water saturation and surface roughness of fabric can affect wetness perception. Therefore, the current study has used fabrics with small surface roughness to reduce the effect of surface texture on wetness perception. Thus, this study is focused on drying time and amount of water in fabric.

In considering the complex mechanism of wet perception and the uncomfortable drying fabric on epidermis, the wet sensation against drying fabrics needs to be investigated. This helps to obtain the overall interaction between wet stimuli and perception. The wet perception assessment is conducted against fabrics at various drying time and different amount of water in fabrics. The mechanical stimulus of the assessment is given by motor-driven fabric movement against participants' forearms. Up to now, there is almost no fabric-based subjective wetness assessment against drying time reported in the literature, but Niedermann *et al*. investigated wet perception based on certain per cent of time to fully dry fabrics [24].

This study aims at investigating wet perception of humans against drying fabrics over time, from wet fabrics to dry fabrics. The drying time dependence of wetness perception is studied. At the same time, the relationship between wet sensation and amount of water per unit volume in fabric is also discussed. Finally, the performance of fabric based on wetness perception and time for fabric to completely dry is compared.

## 2. Methods

### 2.1. Set-up of fabric driver

**Figure 1.**
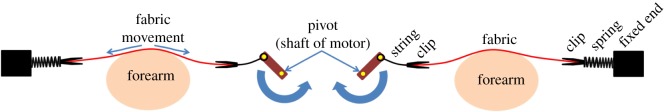
Schematic diagram of two-arm fabric driver for subjective wetness assessment (cross section). The driver was synchronized to drive specimens and reference fabrics on both forearms of the participants.

In order to provide repeatable fabric movement against participants' forearms, a two-arm fabric driver (figure 1) was built to drive fabrics to and fro on participants' forearms. The design of the driver was with reference to the experimental set-up of [25]. However, the driver built for this study moved two fabrics in phase on forearms of subjects. This allowed comparison of wet perception between reference fabric and sample fabric simultaneously. The participants were not required to memorize the perception against the reference fabric, so the possibility of drift in reference value can be avoided. Therefore, participants can focus on comparing the difference between wetness of the specimen and reference. This helped to enhance the reliability of the assessment.

The motion of fabrics was actuated by the rotation of two motor-driven paddles. The paddles pulled fabrics via inelastic strings at the first-half cycle and sent back fabrics by springs at the second-half cycle. Amplitude of fabrics moving across the skin was 2.5 cm. The paddles spent 1.5 s for one revolution. The highest point that paddles reached and the highest point of forearm skin were set at the same height. This controlled pressure applied onto the skin. No vertical pressure was applied externally onto fabrics. Clips were used to hold fabric at around 0.5 cm at both ends. This allowed quick reloading of test and reference specimens in every trial. The specimens were moved along the length of fabrics. This prevented large elongation of knitted fabric during tests. Reference fabric was presented on one of the forearms of the participant; testing fabric was presented on the other forearm of the participant. The left–right arrangement of reference specimen was randomly decided at the beginning of the assessment. The left–right arrangement was fixed for each participant to avoid confusing participants and operators during the tests.

### 2.2. Specimens, reference fabric and environmental conditions

Eight fabrics were used for the subjective wet perception assessment. They were CnP, RAY, COT1, COT2, P3M, WOL, SIL and PET (appendix A). These samples include knitted and woven fabrics with various fibre contents; specifications are listed in table 1. These specimens were of size 12 cm × 12 cm and were gently ironed for a flat surface, and then conditioned under standard atmospheric conditions (temperature 20 ± 1°C and 65 ± 5% relative humidity (RH)) for at least 12 h. The subjective tests were conducted under the same conditions. COT2 carrying 0.8 g of water was selected as the reference fabric. The amount of water was defined empirically. The COT2 for reference was wetted but not yet saturated for comparing with specimens.

**Table 1.** Specifications of fabrics.

fabric code	knitted/woven	fabric structure	fibre content	weight (g m^−2^)	thickness (mm)	absorption capacity (mg cm^−2^)^a^	warp SMD (µm)^b^	weft SMD (µm)^b^
CnP	knit	single jersey	40% cotton, 60% polyester	144.6	0.56	37.2	1.66	1.69
RAY	knit	single jersey	95% rayon, 5% spandex	259.0	0.86	61.5	2.48	2.04
COT1	woven	plain	cotton	56.6	0.37	14.6	3.24	3.64
COT2	woven	plain	cotton	156.9	0.42	18.3	3.28	2.49
P3M	woven	plain	96% plyester, 4% spandex (sport dry fit, 3M)	89.1	0.28	16.5	2.36	2.35
WOL	woven	plain	wool	283.2	0.64	25.3	2.30	5.29
SIL	woven	plain	silk	57.9	0.16	12.4	1.26	2.52
PET	woven	plain	polyethylene terephthalate	66.8	0.12	7.27	1.91	1.45

^a^Method provided in [26]. In the current study, the result is expressed as water absorbed per unit area of fabric.

^b^SMD: mean deviation of fabric surface roughness of fabric measured by KES-FB4 Kawabata automatic surface tester.

All fabrics were wetted by the following procedures. First, 1.4 g of water was sprayed on a plastic card of size 12 cm × 12 cm. Then, each fabric specimen was put on the wetted plastic card, and the stack was pressed at a pressure of 2.5 g cm^−2^ for 5 s. After pressing, the fabric was removed from the plastic card immediately. This ‘stamping’ method allows non-absorbed water to remain on the plastic card. The residual water on the plastic card was disregarded. The amount of absorbed water by fabrics can be found in §3.1 (amount of water at drying time zero minute in figure 3). Disregarding that residual water constitutes actual wearing situation that sweat may roll off if it is not absorbed. 1.4 g of water on 12 cm × 12 cm area (around 10 mg cm^−2^) corresponds to sweat amount for around 23–61 min of light activity (converted and summarized from the literature [27–29]). The pressure (2.5 g cm^−2^) was with reference to Gravimetric Absorbency Testing System [30] for achieving good contact between the fabric and the wetted plastic card. After stamping, the wetted specimens were put for drying on a bench for 0, 16, 32, 48 or 64 min. Once the pre-set drying time was up, the sample was delivered to a participant's forearm for assessment. This is named as the drying time of fabric (DToF).

CnP-00 denotes DToF of 0 min and CnP-16 represents DToF of 16 min; other durations are denoted similarly. The amount of water carried by all specimen fabrics was weighed by an electronic balance (Mettler Toledo, MS1003S; resolution 1 mg, repeatability 1 mg) just before the fabrics were presented to participants for wetness evaluations. As mentioned previously, reference fabric was COT2 which carried 0.8 g of water. The spraying and stamping procedures of reference fabric were the same as specimens; however, around 0.8 g of water was sprayed and DToF was 0 min.

### 2.3. Procedures of the subjective assessment

The participants were asked to confirm if they were in a normal physical condition. Then, detailed instructions on the wetness assessment were given to the participants. The participants gave informed consent and provided their age and sex. After that, participants were asked to wash their forearms with water and then acclimatize the same under standard atmospheric conditions (20 ± 1°C and 65 ± 5% RH) for 30 min.

**Figure 2.**
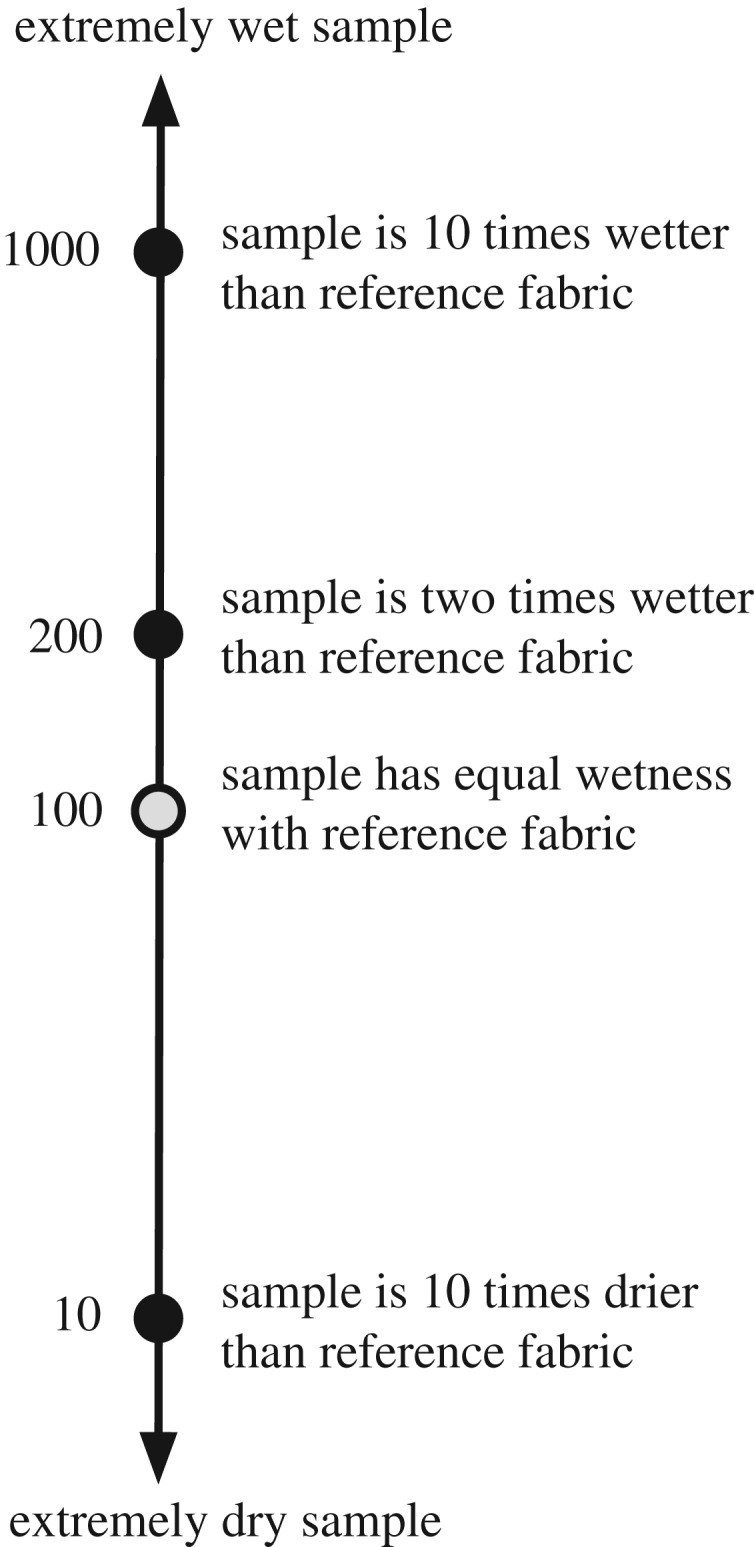
Ratio scale used for the wet perception assessment. In comparing wet perception of sample against reference, wetness rating is given in terms of percentage.

When the training session began, participants were invited to sit on a chair and put both arms on a table. Participants were asked to decide a comfortable sitting posture on their own. A curtain was set in front of participants to ensure that blind tests were conducted. Ten training fabrics were presented to each participant. The training fabrics were CnP-32, RAY-32, COT1-32, COT2-32, P3M-32, WOL-32, SIL-32, PET-00, PET-32 and PET-64. These ten fabrics were presented to participants randomly for familiarizing wet perception rating scale. DToF of 32 min is at the middle of the test protocol, so fabrics at DToF of 32 min are used. Moreover, PET-00 and PET-64 covered a wide range of amount of water per unit volume, and so wetness rating. Each participant was asked to assign wetness rating for all training fabrics. The wet perception rating scale was in ratio and based on the comparison of wet perception of the reference fabric and the specimen. The ratio scale is visualized in figure 2. When reference and specimen were the same in terms of wet perception, wetness rating should be 100 (per cent). If the participant sensed that wetness of the specimen was half of the reference, wetness rating of 50 (per cent) should be given. By contrast, if the participant sensed that the specimen was 10 times wetter than the reference, then 1000 (per cent) rating was to be assigned to a specimen. The wetness rating can be any non-zero positive number, and not limited to integers. Under this rating scale, participants do not need to deal with the meaning of adjectives in ordinal point scale [31]. For example, wordings slightly wet, barely wet, etc. were used in previous studies [32,33]. Therefore, participants can focus on comparing wet sensation between sample and reference in the current study. Moreover, in ordinal point scale, participants may avoid the extremes [34]. Thus, the ratings are confined to the middle of the scale [25,34]. The ratio scale gives no boundary to a participant's rating for comparing wetness perception, so the effect of central tendency in ordinal scale may be suppressed.

In order to prevent drop in skin sensitivity by prolonged exposure under stimulants, participants had 15 s limit to rate each sample. If time was over, the corresponding wetness rating should be left blank. One specimen fabric with one reference fabric was presented to participants every minute. There was at least 45 s rest time between assessments. After each test, participants should get soft tissues to gently absorb residual water on the skin. However, rubbing was prohibited to prevent epidermal irritation [20]. Abovementioned procedures were applied to the whole subjective wetness assessment. Test samples CnP, RAY, COT1, COT2, P3M, WOL, SIL and PET were wetted and then held for 0, 16, 32, 48 and 64 min before being presented to participants. Therefore, each participant made assessment 50 times, including those 10 times under training. All sensory assessments were conducted in a single day for each participant. This prevented within participant drift and contributed to reliability. After sensory test, the wetness rating is projected onto the whole time span of drying process. The projection of wetness rating is able to limit the duration of sensory assessment, so that the possibility of sensory fatigue can be reduced. This shows the need of projection of wetness rating.

### 2.4. Participants and pre-screening test of participants

Twenty-one participants were invited for wet perception assessment. One of them was rejected by the pre-screening test (pre-screening test of participants is specified in the next paragraph). Therefore, 7 male and 13 female participants participated in the assessment. The age range of participants was 23–38, with average age being 28. These participants had no background knowledge about specimens and reference fabric.

The three PET training fabrics mentioned in the last sub-section (PET-00, PET-32 and PET-64) also acted as pre-screening fabrics for participants. PET-00 is fully saturated with water, PET-64 is almost completely dried, and PET-32 is at the midway. If the participant was unable to give wetness rating of these three fabrics in descending (≥) sequence, that participant would have unknown difficulties for the wetness perception assessment. In order to reduce uncertainty throughout this study, that participant was not invited for further analysis. Finally, one participant was not invited for the remaining assessment under this pre-screening criterion. Pre-screening session was also employed to check the eligibility of participants in previous sensory tests [32,35].

### 2.5. Statistical analysis

Nonparametric statistics tests are applied to evaluate the sensitivity of wetness assessment method. The sensitivity of ‘DToF to wetness rating’ and ‘fabric type to wetness rating’ are investigated. Because no nonparametric statistics can test two independent variables in a single test [36], the nonparametric equivalent of two-way ANOVA would not be available. Therefore, Friedman tests are conducted to assess the effect of DToF (0th min, 16th min, 32nd min, 48th min and 64th min) on wet perception. Friedman test is a nonparametric statistical rank test to compare multiple related samples [34,37]. On the other hand, the effect of fabric type on wet perception is tested by Kruskal–Wallis test. Kruskal–Wallis test is a nonparametric statistical rank test to compare multiple unrelated samples [34,37]. IBM SPSS Statistic 22 is used to conduct these statistical tests.

## 3. Results

### 3.1. Amount of water carried by fabric

**Figure 3.**
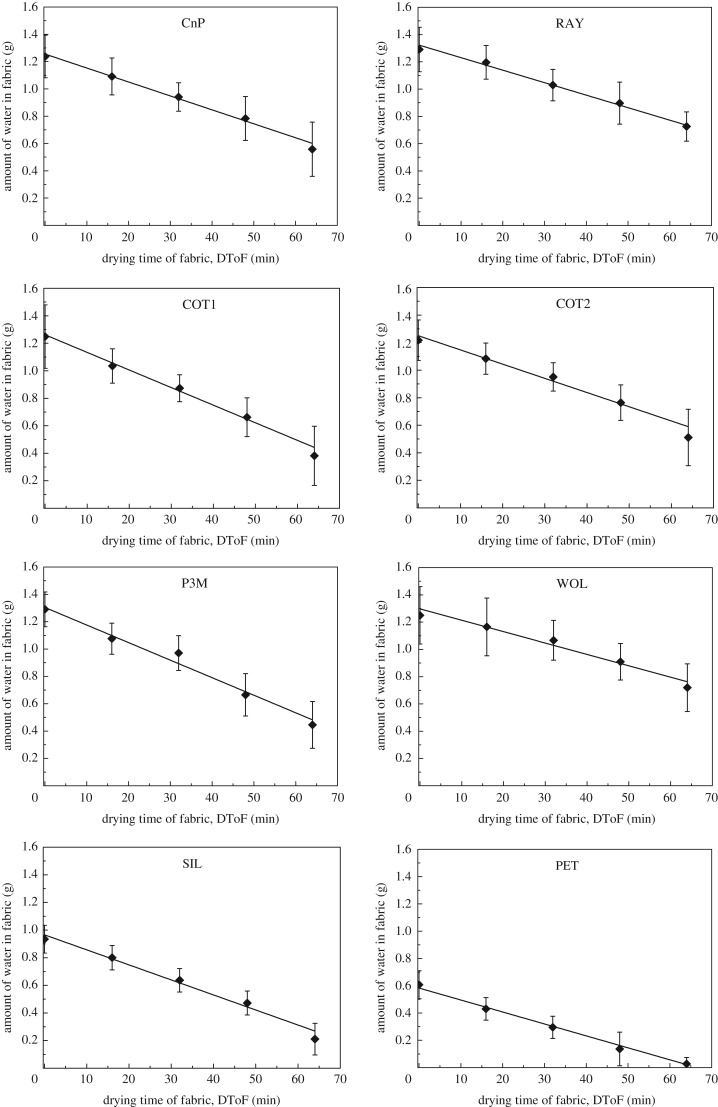
Plots of amount of water in fabric at corresponding DToF (20 participants). Error bars show one standard deviation of uncertainty.

In figure 3, the *y*-axis of plots represents the water carried by fabric before it is delivered onto a participant's forearm. The *x*-axis is the DToF under standard atmospheric conditions. Linear regressions are conducted on each of the plots shown in figure 3. Slope, intercepts and *R*^2^-value of regressions are listed in table 2. The *R*^2^-values of all these eight plots are at least 0.97. This implies that water amount on fabrics decreases constantly under a natural drying process. The slope of plots represents the drying rate of fabrics at standard atmospheric conditions. The *x*-intercept is an extrapolated value that represents the projected time for fabric to be completely dried. The time for completely dried reflects time span of wet perception. This is also a factor that affects comfort of fabrics in real use. As summarized in the Introduction, amount of water per unit volume of fabric is crucial for wet sensation. Amount of water per unit volume is also shown in table 2. It is determined from absolute amount of water in fabric, fabric thickness and sample area (144 cm^2^). The amount of water per unit volume included the factor that of fabric thickness [22,23] or water content [26]. As specified in a previous section (§2.2), ‘stamping’ method was applied to demonstrate the roll off phenomenon of non-absorbed water. The residual water becomes obvious for SIL and PET. Data point 0 min of ‘water amount against DToF’ (figure 3) indicates that SIL and PET carry less than 1.0 g of water after pressing. The ‘water amount against DToF’ plots (figure 3) show different fabric drying rates. PET-64 was almost completely dried at 64th minute. On the other hand, in RAY-64 and WOL-64 roughly only half of the absorbed water was evaporated.

**Table 2.** Slope, intercepts and *R*^2^-value of plots shown in figure 3 (amount of water in fabric against DToF); water per unit volume in fabric (based on value presented in figure 3, fabric thickness and area).

fabric	slope (drying rate, g min^−1^)	*y*-intercept (water carried at 0th minute, g)	*R*^2^	*x*-intercept (expected time for fabric drying out, minute)	average amount of water per unit volume in fabric at ‘xx’ minutes of DToF (mg cm^−3^)				
					0th min	16th min	32nd min	48th min	64th min
CnP	−0.0104	1.26	0.99	120	153	135	117	97	69
RAY	−0.00894	1.31	0.99	147	104	97	83	72	59
COT1	−0.0132	1.26	0.99	95.8	234	194	164	124	72
COT2	−0.0108	1.25	0.98	116	201	179	157	126	85
P3M	−0.0131	1.31	0.98	99.7	320	267	241	165	110
WOL	−0.00823	1.29	0.97	156	136	126	116	99	78
SIL	−0.0111	0.966	0.98	87.0	406	347	276	205	91
PET	−0.00909	0.590	0.99	64.9	352	249	171	79	16

### 3.2. Sensation test result: wetness rating

**Figure 4.**
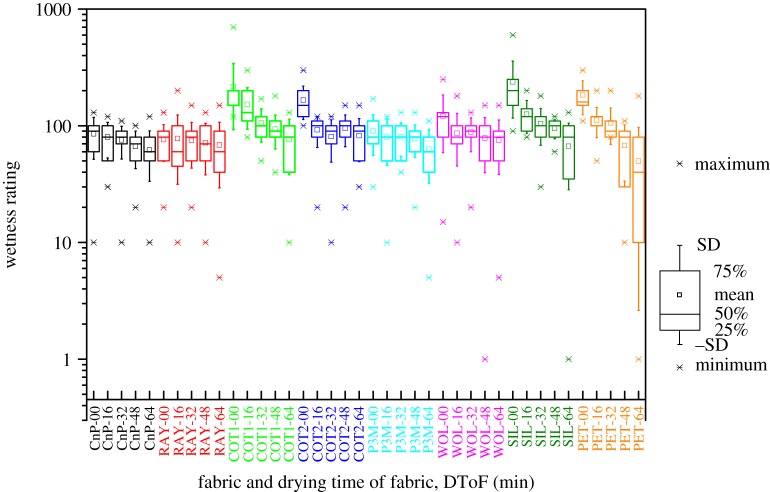
Wetness (perception) rating of all assessed fabrics at various DToF (20 participants). (For example, label ‘RAY-16’ on *x*-axis refers RAY with DToF of 16 min; wetness rating of 1000 (per cent) means specimen is 10 times wetter than the reference; wetness rating of 50 (per cent) refers to wetness of the specimen being half that of the reference.)

Figure 4 is a box-and-whisker plot of subjective wetness ratings of all tests. The fabrics and DToF are labelled on the *x*-axis. A base-10 logarithmic scale is used in the *y*-axis. This is because the range of wetness rating is not limited, and a wide range of wetness ratings (from 1 to 700) was given by the 20 participants. In the box-and-whisker plot, a small square indicates the mean of wetness ratings. The bottom and top of the box show 25 percentile and 75 percentile of data, respectively. The band inside the box is the median of the results. Maximum and minimum of data are marked with crosses. Finally, the whiskers represent one standard deviation apart from the average value.

The drop in wetness rating against DToF changes is obvious in cases of relatively thin specimens (COT1, SIL and PET). Wetness rating of COT1-00 and SIL-00 was higher than 500 by some participants. This is because these specimens are saturated with water during such wetness assessment, and water is in contact with participants’ forearm intimately.

### 3.3. Categorization of wetness rating results

**Figure 5.**
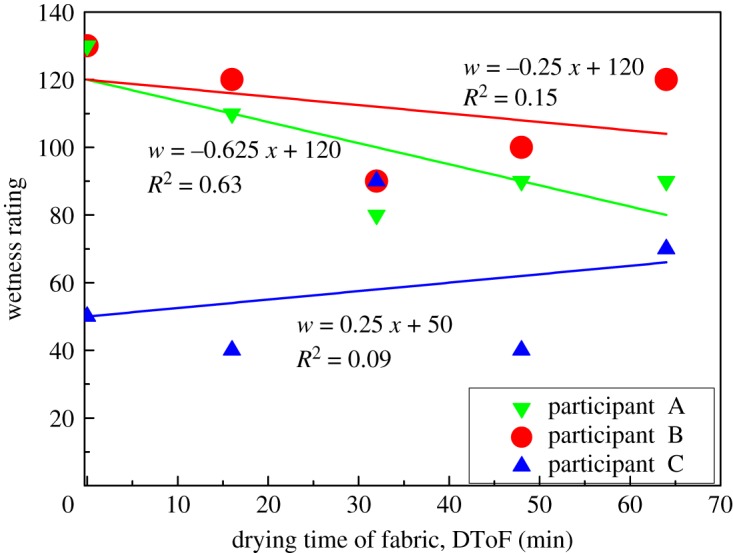
Wetness rating against DToF of CnP of participants A, B and C. Participant A sensed drop in wetness (*R*^2^ ≥ 0.50 and a negative slope). Participants B and C do not sense change in wetness (*R*^2^ < 0.50).

Figure 5 presents three sets of data of CnP from three individual participants. Each set of data consists of five data points. The data point refers to wetness rating at the corresponding DToF of a fabric rated by a participant. These examples help to introduce the rules of categorizing each set of data. Participants' wetness perceptions are defined into two categories. Category (I): drop in fabric wetness can be sensed by participant when the fabric is drying. Category (II): change in fabric wetness cannot be sensed by participant when the fabric is drying.

The first set of data of CnP (figure 5, participant A) shows a participant sensed lower wetness when drier fabrics (larger DToF) were presented. When linear regression of a set of data gives *R*^2^ ≥ 0.50 and a negative slope, this set of data is defined as Category (I) where the drop in fabric wetness can be sensed by participant when the fabric is drying. Some fluctuations in data are accepted in subjective sensation test, e.g. 32nd minute shown in figure 5. Category (I) fabrics are listed in table 3 which also lists the average value of slope and the average of *y*-intercept of ‘wetness rating against DToF of each participant’. It should be noted that dataset with *R*^2^ ≥ 0.50 and positive slope should be rejected. Positive slope reflects that the participant may be unstable for wetness assessment. Therefore, all assessment results given by that participant should be disregarded. No rejection of a participant is recorded at data analysis stage.

**Table 3.** Datasets that fall into Category (I): drop in fabric wetness can be sensed by participant when the fabric is drying.

fabric	number of datasets (participants) that fall in Category (I)	average	standard deviation	coefficient of variation	average	standard deviation	coefficient of variation
		of slope of ‘wetness rating against DToF’			of *y*-intercept of ‘wetness rating against DToF’		
CnP	10	−0.644	0.236	0.37	98.8	13.0	0.13
RAY	1	−0.438	—	—	104	—	—
COT1	14	−2.57	1.81	0.70	213	99	0.46
COT2	11	−1.22	0.71	0.58	138	30	0.22
P3M	6	−0.828	0.349	0.42	121	17	0.14
WOL	8	−1.21	0.69	0.57	128	23	0.18
SIL	19	−2.45	1.57	0.64	206	71	0.34
PET	18	−2.02	1.06	0.52	165	46	0.28

When linear regression of a dataset gives *R*^2^ < 0.50, such set of data is regarded as Category (II) where the change in fabric wetness cannot be sensed by participant when the fabric is drying. This implies a no significant trend in wetness perception when fabrics with different DToFs are assessed. Therefore, both positive and negative slopes in linear regression are included in Category (II); examples can be found from two sets of data of CnP (figure 5, participants B and C).

Table 4 summarizes the number of datasets that fall into Category (II) and average wetness ratings at various DToFs. Participants' perceptions against CnP, RAY, P3M and WOL mainly fall into Category (II). Their average wetness ratings are rather constant with respect to DToF (table 4). The wetness ratings listed in table 4 also reflect that the participants feel that CnP, RAY and P3M are drier than WOL. In contrast with CnP, RAY, P3M and WOL, averaged Category (II) wetness ratings of COT1, COT2, SIL and PET are not constant with respect to DToF.

**Table 4.** Datasets that fall into Category (II): change in fabric wetness cannot be sensed by participant when the fabric is drying.

fabric	number of datasets (participants) that fall in Category (II)	average wetness rating at ‘xx’ minutes of DToF (standard deviation)				
		0th min	16th min	32nd min	48th min	64th min
CnP	10	70 (38)	73 (28)	71 (28)	69 (21)	64 (32)
RAY	19	75 (26)	77 (46)	74 (32)	71 (33)	68 (39)
COT1	6	182 (62)	130 (60)	98 (27)	105 (43)	107 (18)
COT2	9	178 (54)	74 (29)	82 (31)	108 (25)	99 (31)
P3M	14	78 (29)	68 (32)	74 (23)	74 (16)	63 (32)
WOL	12	110 (70)	83 (51)	83 (33)	92 (40)	86 (39)
SIL	1	90 (/)	80 (/)	100 (/)	100 (/)	80 (/)
PET	2	215 (/)	105 (/)	155 (/)	65 (/)	130 (/)

### 3.4. Relationship between wetness rating and drying time of fabric

**Figure 6.**
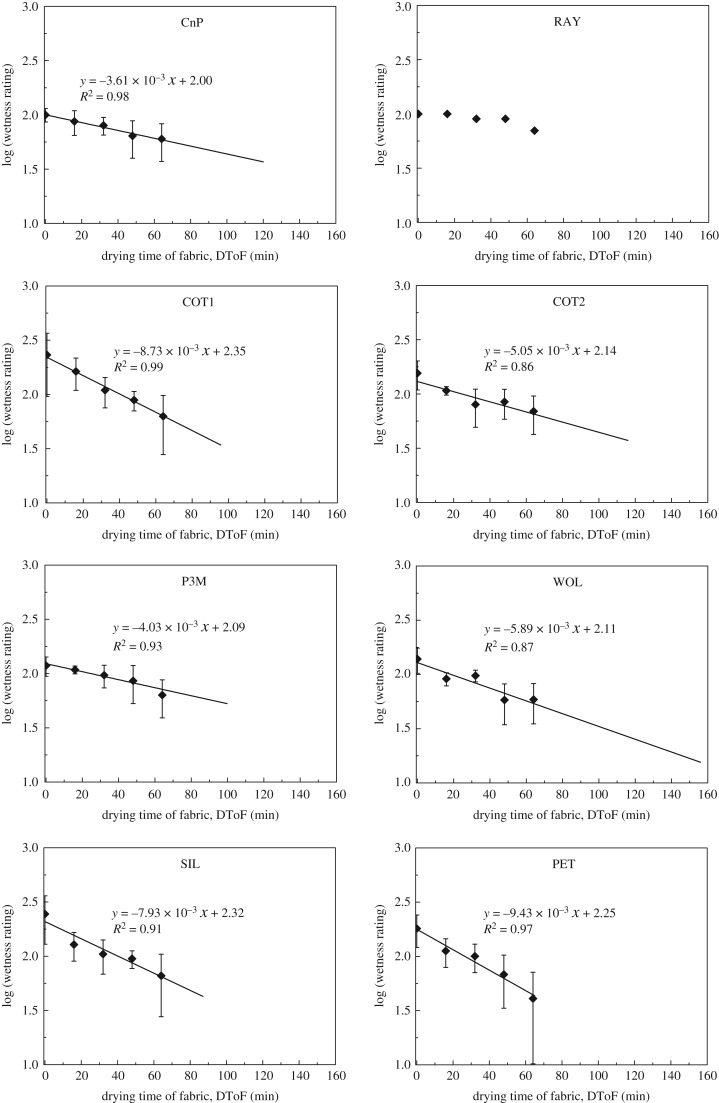
Semi-log plots of wetness rating (average of Category (I) perception result) against DToF. Error bars show 1 s.d. of uncertainty. Linear best-fits are extrapolated to the DToF at fabrics completely dried.

Based on datasets that fall into Category (I) (column 2 of table 3), the analysis is further conducted to compare results of different fabrics and to project wetness rating. Line-fitting and then projection are done for wetness rating from fabrics freshly wetted (0th min) until dried completely. The time of fabric drying out is obtained from the *x*-intercept shown in ‘water amount on fabric at corresponding DToF’ (figure 3, also listed in the fifth column of table 2). Plots ‘wetness rating against DToF’ according to Category (I) are shown in figure 6 as semi-log plots. For example, in figure 6, 10 results (from 10 trials assessed by 10 participants) of CnP-00 are averaged, and then take log_10_ to be the first data point. The log_10_ operation enables the conduction of linear regression on the log(wetness rating) and DToF relationship. The error bar shows one standard deviation of uncertainty. The plots shown in figure 6 help to study the relationship between wetness rating and DToF. This represents the wetness perception against wetted fabrics while fabrics are drying.

**Figure 7.**
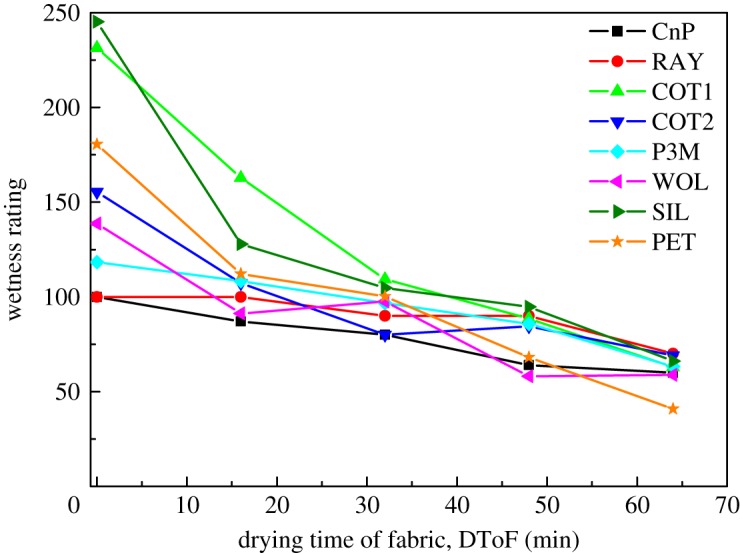
Wetness rating against DToF of Category (I) perception results. Wetness rating converges into a small range when fabrics approach dryness.

As shown in table 3 ‘datasets that fall into Category (I)’, RAY has only 1 set of data that falls in Category (I) perception. Therefore, only data are presented and no line-fitting is shown in the plots of ‘wetness rating against DToF’ in figure 6. All other seven tested fabrics (CnP, COT1, COT2, P3M, WOL, SIL and PET) have good linear fit between log(wetness rating) and DToF (figure 6). Log(wetness rating) and DToF are correlated by linear regression. This gives very high *R*^2^-values (0.86–0.99) to these seven fabrics. In summary, wetness perception has a logarithmic relationship with DToF. Figure 7 visualizes and summarizes wetness rating against DToF in linear scale.

### 3.5. Relationship between wetness rating and amount of water in fabrics

**Figure 8.**
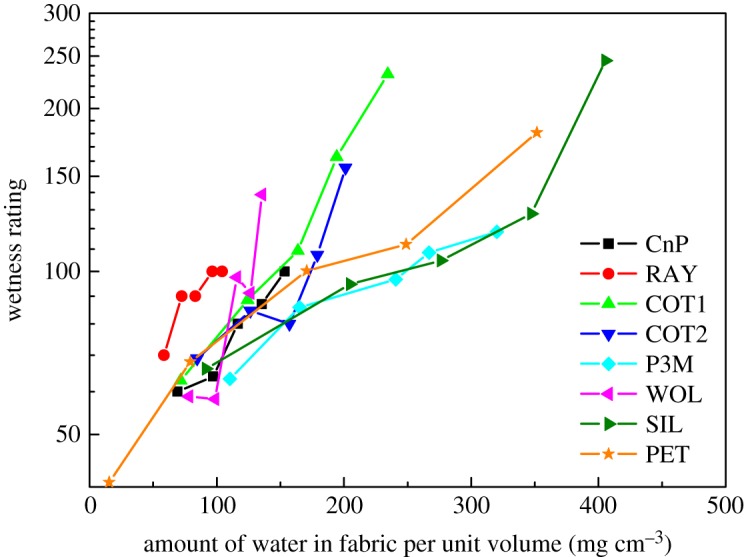
Wetness rating against amount of water per unit volume in fabric of Category (I) perception results; *y*-axis is in log scale.

Under Category (I) perception, figure 8 shows a semi-log plot ‘wetness rating against amount of water per unit volume’ in fabric. This plot presents roughly linear relationship for each of the fabrics. Rate of change of wetness rating goes high when the amount of water per unit volume increases. The slope of P3M, SIL and PET is slightly lower than that of other fabrics. The finding noted in figure 8 matches with previous studies [22,23]. It indicated that wetness perception relates to fabric saturation values.

### 3.6. Within-participants reliability

The 20 participants that passed the pre-screening test were invited to conduct full wetness perception tests. By applying the rejection criterion mentioned in §3.3 (a set of data with *R*^2^ ≥ 0.50 and positive slope in linear regression), none of the 20 participants was rejected. Therefore, all these participants give consistent results in each set of data. ‘Each set’ of data refers to five wetness rating data at various DToFs of the same fabric. As participants give consistent results, they are reliable for the subjective assessment.

### 3.7. Between-participant consistency

Datasets defined as Category (I) refer to participants’ sensing drop in wetness when the fabric is drying. Each set of data gives slope and *y*-intercept. Average value of slope and *y*-intercept of each fabric gives coefficients of variation (CVs) (table 3). CVs of the slope of fabrics range from 0.37 to 0.70. CVs of *y*-intercept of fabrics are between 0.13 and 0.46. The CVs are within acceptable range. The variations depend on the sensitivity of participants. This is because a more sensitive participant gives results with larger slope and *y*-intercept. Therefore, the between-participant consistency is reasonable in this study.

### 3.8. Sensitivity of wetness assessment

#### 3.8.1. Effect of drying time of fabric on wet perception

As summarized in tables 5 and 6, under Category (I), there are seven individual Friedman tests conducted against seven fabrics (CnP, COT1, COT2, P3M, WOL, SIL and PET). The null hypotheses of Friedman tests are ranks of wetness ratings at all DToFs (0th min, 16th min, 32nd min, 48th min and 64th min) are the same. The mean ranks of wetness rating at ‘0th min’ are the largest (table 5). This represents that the wetness rating at ‘0th min’ is the highest in terms of DToF. All seven null hypotheses are rejected at 0.050 significance level. Therefore, the wetness perception is sensitive to the change in DToF.

**Table 5.** Seven individual sets of wetness ratings by ranks for Friedman tests against DToF. Type of fabric is the fixed factor.

	mean rank of wetness rating						
	fabric						
DToF	CnP	COT1	COT2	P3M	WOL	SIL	PET
64th min	1.70	1.39	1.68	1.08	1.81	1.24	1.31
48th min	2.15	2.14	2.55	2.42	1.38	2.42	2.11
32nd min	2.90	2.82	2.27	3.33	3.63	2.84	3.11
16th min	3.70	3.79	3.73	3.67	3.44	3.61	3.61
0th min	4.55	4.86	4.77	4.50	4.75	4.89	4.86

**Table 6.** Test statistics of Friedman tests of seven fabrics.

fabric	CnP	COT1	COT2	P3M	WOL	SIL	PET
*N*	10	14	11	6	8	19	18
*χ*^2^	23.0	43.2	29.9	18.0	26.1	59.6	57.9
d.f.	4	4	4	4	4	4	4
asymp. sig.	0.000	0.000	0.000	0.001	0.000	0.000	0.000

#### 3.8.2. Effect of fabric type on wet perception

Five Kruskal–Wallis tests are conducted with the DToF (0th min, 16th min, 32nd min, 48th min and 64th min) as the fixed parameter in each test. The null hypothesis of each test is that all fabrics give equal rank in wetness rating. The mean rank of wetness rating (table 7) indicates the differences in wetness perception against various fabrics. The higher mean rank refers to higher wetness rating. It is found that at 0th min, 16th min and 48th min, the null hypotheses are rejected at 0.050 significance level (table 8). Therefore, the wetness rating is sensitive to fabrics at DToF of 0th min, 16th min and 48th min. On the other hand, the null hypotheses are accepted at 32nd min and 64th min.

**Table 7.** Five individual sets of wetness rating by ranks for Kruskal–Wallis tests against fabric. DToF is the fixed factor. *N*: number of participants.

fabric		mean rank of wetness rating				
		DToF				
	*N*	0th min	16th min	32nd min	48th min	64th min
CnP	10	9.70	22.2	26.0	27.9	43.6
COT1	14	59.1	67.3	54.0	50.2	46.3
COT2	11	38.9	39.6	33.7	49.7	50.8
P3M	6	19.1	40.8	44.3	49.8	47.2
WOL	8	31.7	21.6	45.6	21.6	42.8
SIL	19	61.8	51.6	50.4	56.3	48.3
PET	18	47.1	41.4	42.6	37.3	30.9
total	86

**Table 8.** Test statistics of Kruskal–Wallis tests of the five DToF. Type of fabric is the grouping variable.

	wetness rating				
	0th min	16th min	32nd min	48th min	64th min
*χ*^2^	43.4	29.3	10.8	18.6	6.59
d.f.	6	6	6	6	6
asymp. sig.	0.000	0.000	0.093	0.005	0.360

## 4. Discussion

Fabric wetness rating is determined by participants at various DToFs. The wetness rating represents the actual perception against the wetted fabric. The time dependence of wetness rating shows wet perception against drying fabrics over time. The discussion focuses on the relationship between the wetness rating, DToF and amount of water in fabric. Comparison between fabric wetness performance is also provided.

### 4.1. Relationship between wetness rating and drying time of fabric

The wetness rating recorded from 20 participants is consistent with the general understanding that wet sensation decreases with the increase of DToF (figure 4). The rate of decrease of thin fabrics is higher than that of thick fabrics. This is because the thin fabrics (COT1, SIL and PET) present wide ranges of amount of water per unit volume in fabrics (table 2). For example, the amount of water in PET drops from 352 mg cm^−3^ to 16 mg cm^−3^ from 0th min to 64th min. This observation matches with pervious works on the effect of fabric thickness on wetness sensation [22,23].

#### 4.1.1. Difference between Category (I) and (II) perceptions

Category (II) perception refers to no sense of change in wetness of a fabric type at different DToF. The no sense in wetness is understandable because the change in fabric's wetness level is too small. The wetness level can refer to the amount of water per unit volume in fabric at different DToF (table 2). CnP, RAY, P3M and WOL mainly fall into Category (II). Their wetness rating in Category (II) is independent of the change of DToF (table 4). This is coherent with the definition of Category (II) perception ‘no sense in change’. In addition, results of some other fabrics (e.g. SIL and PET mostly fall into Category (I)) show that participants are sensitive to wetness change of fabric (§3.8). This shows the number of datasets that fall into the categories because of fabric properties, but not because of the sensitivity of participants.

#### 4.1.2. Sensitivity of fabric type on wet perception

As mentioned in §3.8.2, two of the Kruskal–Wallis tests accept the null hypotheses. This means that fabric type cannot be distinguished by wetness rating at DToF of 32nd min and 64th min. This is because the wetness perceptions are similar when fabrics are almost dried out. The wetness rating converges into a small range. The trend of convergence in wetness rating can be observed in figure 7. The similar wet perception when fabric dried out was also observed in [24]. In other words, the wetness perception assessment is sensitive to fabric types.

### 4.2. Comparing wetness performance among fabrics

#### 4.2.1. Using the result of Category (I) perception

As the semi-log relationship represents the interaction between wetness rating and DToF, it is used to compare wetness performance of fabrics. Equations of the semi-log relationship (figure 6) are employed for analysis. Log(wetness rating) of fabrics can be found by substituting the required DToF into the equations. Therefore, the wetness ratings of fabrics at any DToF in the drying process are known. This feature offers a quick solution for researchers and product developers to study wet sensation against fabric.

Table 9 lists the key results of fabric wetness performance throughout the following comparison. The comparison begins with substituting DToF = 0 and DToF = ‘time for fabric dries out’ (5th column of table 2) into equations shown in ‘semi-log plots of wetness rating against DToF’ (figure 6). The results give the range of projected wetness rating in Category (I) perceptions. The range of each fabric is listed in Column 2 of table 9. The projected wetness rating of CnP, COT1, COT2, P3M, SIL and PET converges into a closed value from 33 to 49 when fabrics are dried. The projected wetness rating of WOL when the fabric dries out is 16. A similar value of projected wetness rating, when fabric dries out, supports that the projection is reasonable.

**Table 9.** Key parameters obtained against wetness performance of fabrics.

	Category (I) perception			range of wetness rating in Category (II) perception, from 0th to 64th minutes^b,c^
	range of projected wetness rating from just wetted to dry out ^a,c^	expected time for fabric dry out (minute)	wetness factor (WF) (dimension: wetness × time)	
source	calculated from equations given in semi-log plot of wetness rating against DToF (figure 6)	*x*-intercept of water amount on fabric against DToF (table 2)	calculated from equations given in semi-log plot of wetness rating against DToF (figure 6)	summary table of Category (II) wetness rating (table 4)
fabric				
CnP	[100, 37) feel: wet → dry	120	7590	[64, 73]
RAY	—	147	—	[68, 77]
COT1	[224, 33) feel: very wet → dry	95.8	9510	—
COT2	[138, 36) feel: wet → dry	116	8790	—
P3M	[123, 49) feel: wet → dry	99.7	8000	[63, 78]
WOL	[129, 16) feel: wet → dry	156	8350	[83, 110]
SIL	[209, 43) feel: very wet → dry	87.0	9110	—
PET	[178, 43) feel: very wet → dry	64.9	6190	—

^a^[inclusive value, exclusive value).

^b^[inclusive value, inclusive value].

^c^Column 2 shows Category (I) perception from just wetted to dry out, and column 5 lists Category (II) perception of first 64 min. The values in these two columns should not be compared directly, but column 5 can be regarded as additional information to column 2.

Moreover, by integrating equation (B3) with respect to *x* (DToF), with limits between 0 and ‘time for fabric drying out’, the area under the curve represents the combined effect of user suffering a wet perception and the time span of wet perception. This total suffering from wet and time is quantified as wetness factor (WF) with a dimension of wetness × time. For example, one fabric has high wetness rating and dries fast, another fabric has low wetness rating and dries slow. Therefore, it is difficult to compare the total suffering (wetness × time) between fabrics. However, WF (the area under curve) provides relative comparison between fabrics. A smaller WF indicates that the user suffers less against wetness. The calculation of WF of fabric CnP is shown in appendix B as an example.

WF of CnP is 7590, WF of the other six fabrics with Category (I) perception is also shown in column 4 of table 9. WF of the seven fabrics falls between 6190 and 9510. COT1 and P3M show a good example of the use of WF results. COT1 and P3M have almost the same time for fabric to dry out (COT1: 96 min, P3M: 100 min; from table 9, column 3). On the other hand, COT1 has high wetness rating when it is just wetted and drops quickly from 224 to 33 (table 9, column 2). P3M has lower wetness rating than COT1 at just wetted, but higher wetness rating near drying out (wetness rating of P3M from 123 to 49). Therefore, the performance of wet perception of COT1 and P3M cannot be distinguished by drying time and the range of wetness rating. However, the WF represents resultant effects of total suffering against wetness by using the area under curve. P3M has WF of 8000, better than COT1 that has WF of 9510. In addition, some of the fabrics show there is a compromise between ‘dries fast’ and ‘offers dry perception’. For example, COT2 and SIL have similar WF values, 8790 and 9110, respectively. COT2 offers dry sensation and water evaporates slowly, but SIL gives wet perception and evaporates fast (table 9, columns 2 and 3). This shows a good fabric for keeping thermal-wet comfort sense should get dried fast and offer dry perception. PET has the smallest WF, 6190. This is because PET has the advantage that it picks up only around half the amount of water compared to other fabrics during the wetting process. This is because of the low water absorption capacity of PET (table 1; 1.05 g for 144 cm^2^ of fabric; 1.05 g lower than the sprayed amount 1.4 g in this wetness assessment). PET takes the least time to dry out, so it gives the shortest wet sensation to participants.

#### 4.2.2. Using the result of Category (II) perception

Column 5 of table 9 shows the range of wetness rating under Category (II) perception (participant does not sense change in wetness when fabric is drying). The range given in Category (II) only represents the wet perception in first 64 min. Only four fabrics (CnP, RAY, P3M and WOL) mainly fall into Category (II). The following example shows the use of Category (II) results. RAY does not have a WF to evaluate its wetness performance directly. The Category (II) perception can help to indicate its performance. According to the discussion in the last paragraph, wet sense and its time span are both important. RAY has Category (II) results similar to CnP (last column of table 9) and longer time to dry out (column 3 of table 9); RAY has similar time to dry out to WOL and better Category (II) wet perception rating than WOL. So, the wetness performance can be stated as CnP (best) > RAY > WOL.

### 4.3. Relationship between wetness rating and amount of water in fabrics

Under Category (I) perception, log(wetness rating) has linear relationship with the amount of water per unit volume in fabric (figure 8). This agrees with the previous finding that wetness perceptions relate to fabric saturation values [22,23]. When the amount of water in fabric per unit volume is below 150 mg cm^−3^, the wetness rating is almost independent of fabric type. This also matches with conclusion drawn by Raccuglia *et al*. [22]. When the amount of water exceeds 150 mg cm^−3^, cotton fabrics (COT1 and COT2) have higher wetness rating than three other fabrics (P3M, SIL and PET).

### 4.4. Limitations

The major limitation of this subjective test is the whole fabric drying process is not conducted on participants' forearms. However, if wetted fabric specimen is put on forearm during the whole drying process, the duration will be at least 10 min for each test. This extended exposure to stimulants is prone to participant fatigue which can affect the reliability of the assessment. This is why five identical specimens with different DToF (0, 16, 32, 48 and 64 min) are presented to participants separately to give breaks and shorten the aggregate test time. Therefore, it is assumed that participants are not fatigued during the assessment. After the training session of 10 fabrics, skin wetness and temperature are assumed to be in steady state.

## 5. Conclusion

The relationship between wet perception and drying fabric is studied. A good linear relationship between log_10_ (wetness rating) and DToF is found for Category (I) perception. Similarly, log_10_ (wetness rating) also has linear relationship with the amount of water per unit volume in fabric. In practical use, WF is introduced to represent the level and the duration of wet perception. A larger WF indicates that the participant (or end user) suffers deeper wet perception when contacting the fabric. This might help fabric researchers and manufacturers in improving fabric that can deliver less wet sensation to end users. WF ranges from 6190 to 9510 among the tested fabrics. In addition to WF, other key parameters obtained in this subjective wet perception study of eight fabrics are summarized in table 9.

## Data accessibility

The parameters given in tables 2–9 are obtained and analysed from experimental data shown in figures. This study has no additional data.

## Appendix A. Nomenclature

**Table:**

WF	wetness factor
DToF	drying time of fabric
fabric codes:	
CnP	cotton and polyester
RAY	rayon
COT1	cotton#1
COT2	cotton#2
P3M	polyester (sport dry fit, 3M)
WOL	wool
SIL	silk
PET	polyethylene terephthalate

## Appendix B. Wetness factor

The calculation of WF of fabric CnP is shown as below.

From figure 6,

B 1 y=−0.00361 x+2.00,

where $y=\;\text{lo}{\text{g}}_{10}(w),w$ is wetness rating, *x* is DToF. Therefore

B 2 $$\text{lo}{\text{g}}_{10}(w)\;=-0.00361\;x+2$$

B 3 $$w=10^{-0.00361\;x\;+2}.$$

Integrating equation (B 3) with respect to *x*, with limit of 0–120 min (*T* is the time for fabric to dry out) gives WF. Note that WF is in terms of area under curve of equation (B 3).

B 4 $$\text{WF}=\int_{0}^{T}w\;dx=\int_{0}^{120}10^{-0.00361\;x\;+2}\text{d}x$$

B 5 $$=10^{2}\int_{0}^{120}10^{-0.00361x}\text{d}x.$$

Let u=−0.00361x, then du/dx=−0.00361. Therefore,

B 6 $$\text{WF}=10^{2}\int_{0}^{-0.00361\;\times \;120}10^{u}\left(\frac{\text{d}u}{-0.00361}\right)$$

B 7 $$=\frac{10^{2}}{-0.00361}\int_{0}^{-0.00361\;\times \;120}10^{u}\text{d}u.$$

By looking up integral table [38], $\int a^{z}\text{d}x=a^{z}/\text{ln( }a)+\text{C},\;a>0,\;a\neq 1,$ and applying it to equation (B 7) gives equation (B 8)

B 8 $$\text{WF}=\frac{10^{2}}{-0.00361}{\left[\frac{10^{u}}{\text{ln(}10)}\right]}_{0}^{-0.00361\times 120},$$

where WF = 7.59 × 10^3^ (unit: minute).

Dimension of WF is wetness × time, and wetness is dimensionless.
